# Insights into the Functions, Characteristics, and Mechanisms of Disease-Related Proteins from *Akkermansia muciniphila*: A Review

**DOI:** 10.3390/microorganisms14040820

**Published:** 2026-04-02

**Authors:** Yanping Han, Juane Lu, Xueying Bu, Liying Hu, Changcheng Niu, Jianjun Qiao, Hao Wu, Qinggele Caiyin

**Affiliations:** 1 State Key Laboratory of Synthetic Biology, School of Synthetic Biology and Biomanufacturing, Tianjin University, Tianjin 300354, China; 2 Zhejiang Institute of Tianjin University (Shaoxing), Shaoxing 312300, China; 3 Key Laboratory of Systems Bioengineering, Ministry of Education (Tianjin University), Tianjin 300072, China

**Keywords:** *Akkermansia muciniphila*, functional proteins, disease regulation, signaling pathways, clinical application

## Abstract

As a representative next-generation probiotic, *Akkermansia muciniphila* (*A. muciniphila*) produces a variety of functional proteins that play critical roles in the prevention and treatment of multiple diseases, including metabolic disorders, inflammatory diseases, neurological disorders, and cancer. This review summarizes the disease-associated proteins of *A. muciniphila* reported to date, including the outer membrane proteins Amuc_1100 and Amuc_1098, as well as the secreted proteins P9 (Amuc_1631), P5, Amuc_1409, Amuc_1434, and Amuc_2109. These proteins exert their biological effects by activating multiple signaling pathways, such as Toll-like receptor 2 (TLR2), ICAM-2, and Wnt/β-catenin, thereby regulating physiological processes including glucagon-like peptide-1 (GLP-1) secretion, serotonin biosynthesis, lipid metabolism, and intestinal stem cell proliferation. This review provides a theoretical foundation and future perspectives for in-depth research investigation and clinical application of *A. muciniphila* disease-related proteins.

## 1. Introduction

*Akkermansia muciniphila* (*A. muciniphila*) is a Gram-negative, strictly anaerobic bacterium that widely colonizes the mucus layer of the cecum and colon in vertebrates, where it utilizes mucin as its sole source of carbon and nitrogen [1]. First isolated from human fecal samples by Derrien et al. in 2004 [2], *A. muciniphila* was the first successfully cultured gut bacterium within the phylum *Verrucomicrobia*. In recent years, owing to its prominent roles in enhancing host metabolic function, maintaining immune homeostasis, and promoting intestinal health, *A. muciniphila* has been widely recognized as one of the most promising next-generation probiotics.

*A. muciniphila* is a key intestinal symbiont that regulates host metabolism and intestinal immunity, and its physiological functions are closely associated with obesity, type 2 diabetes, inflammatory bowel disease and other diseases. Most traditional studies have focused on model strains, whereas recent phylogenomic evidence has revealed that *A. muciniphila* is not a genetically homogeneous group but exhibits substantial strain-level diversity within the species. According to phylogeny and pan-genome analyses, *A. muciniphila* can be divided into several phylogenetic groups. Distinct functional differentiation exists among different strains in terms of genome composition, mucin-degrading capacity, oxygen tolerance, and outer membrane protein structure. Such diversity directly determines the intestinal colonization advantage, metabolic regulatory efficiency, and immunomodulatory activity of each strain, demonstrating typical strain-specific effects. Currently, there are approximately 215 registered strains and 234 isolates in public databases. However, only 5 *Akkermansia* strains (*Akkermansia muciniphila* ATCC BAA-835^T^; AM06; AKM Lab-01^®^; YL-44; KGMB01888) have been formally approved and universally recognized as having completed a comprehensive and systematic evaluation [3,4].

Strain-specific variability further complicates interpretation, as certain human-derived *A. muciniphila* isolates exert protective effects in DSS models while others exacerbate colitis, or fail to demonstrate efficacy [5]. This heterogeneity underscores that *A. muciniphila* cannot be universally categorized as “probiotic” or “pathobiont” but rather represents a microbial “opportunist” whose pathogenic or protective potential is exquisitely sensitive to the tripartite interplay of host genetics, immune status, and environmental factors (diet, inflammation, microbial community context) [6].

With advances in microbiome research, it has become increasingly clear that the health benefits of *A. muciniphila* are not solely attributable to the presence of live bacteria, but are also mediated by functional proteins produced by this microorganism. These proteins play central roles in host–microbe interactions by directly interacting with host cells or modulating the intestinal microenvironment. In particular, the landmark study by Plovier et al. in 2017 [7], which identified the outer membrane protein Amuc_1100, marked a turning point in the field and triggered growing interest in the functional protein repertoire of *A. muciniphila*. Since then, an increasing number of disease-associated proteins [8,9] have been identified and characterized [10,11], providing important mechanistic insights into the health-promoting effects of *A. muciniphila* and offering new opportunities for therapeutic development.

This review summarizes current advances in the identification and functional characterization of disease-associated proteins derived from *A. muciniphila*, analyzes their molecular mechanisms of action, evaluates their therapeutic potential across different disease contexts, and discusses their prospects for clinical translation, with the aim of providing a reference for future research and industrial applications.

## 2. Discovery and Functions of Disease-Associated Proteins from *A. muciniphila*

**Figure 1:**
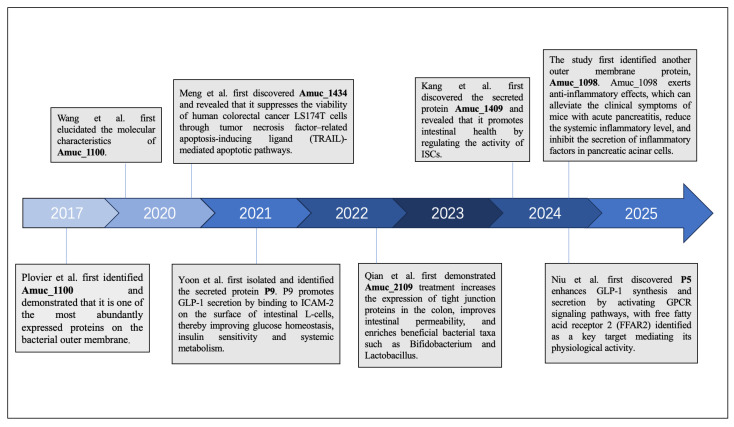
Timeline of discovery and functions of disease-associated proteins (Amuc_1100 [7,13]; Amuc_1098 [14]; Amuc_1434 [9]; Amuc_1409 [11]; Amuc_2109 [10]; P9 [8]; P5 [12]) from *A. muciniphila*.

Currently identified disease-associated proteins from *A. muciniphila* include outer membrane proteins (Amuc_1100 [7] and Amuc_1098) and secreted proteins (P9 [8], Amuc_1409 [11], and P5 [12]). These proteins play prominent roles in regulating glucose and lipid metabolism, enhancing intestinal barrier function, suppressing inflammatory responses, and promoting neuroprotective effects (Figure 1).

### 2.1. Amuc_1100: A Multifunctional Outer Membrane Protein

Amuc_1100 is the most extensively studied protein derived from *A. muciniphila* and has been renamed the pili-associated signaling (PAS) protein [15] (Table 1). Research on *A. muciniphila* outer membrane proteins began in 2017, when Plovier et al. first identified Amuc_1100 and demonstrated that it is one of the most abundantly expressed proteins on the bacterial outer membrane [7]. This discovery provided the first direct evidence that the health benefits of *A. muciniphila* can be mediated by specific protein components, representing a milestone in the field (Figure 1). Amuc_1100 was shown to be a heat-stable, pilus-like protein that retains biological activity and health-promoting effects even after pasteurization.

Subsequent studies further elucidated the molecular characteristics of Amuc_1100 (Figure 1). Structural analyses revealed that its extracellular domain consists of four antiparallel β-strands and four α-helices, forming a core structure composed of two “αββ” motifs, which share structural similarity with type IV pili and type II secretion system proteins [13]. Interestingly, although Amuc_1100 exists as a monomer in solution, it forms trimers in the crystalline state, suggesting that its variable oligomerization may be associated with functional regulation.

As an outer membrane protein with a molecular weight of approximately 33 kDa, Amuc_1100 exerts pleiotropic biological functions, playing critical roles in metabolic regulation [16], immune modulation [17], and intestinal protection [18].

#### 2.1.1. Amuc_1100 and Metabolic Regulation

**Figure 2:**
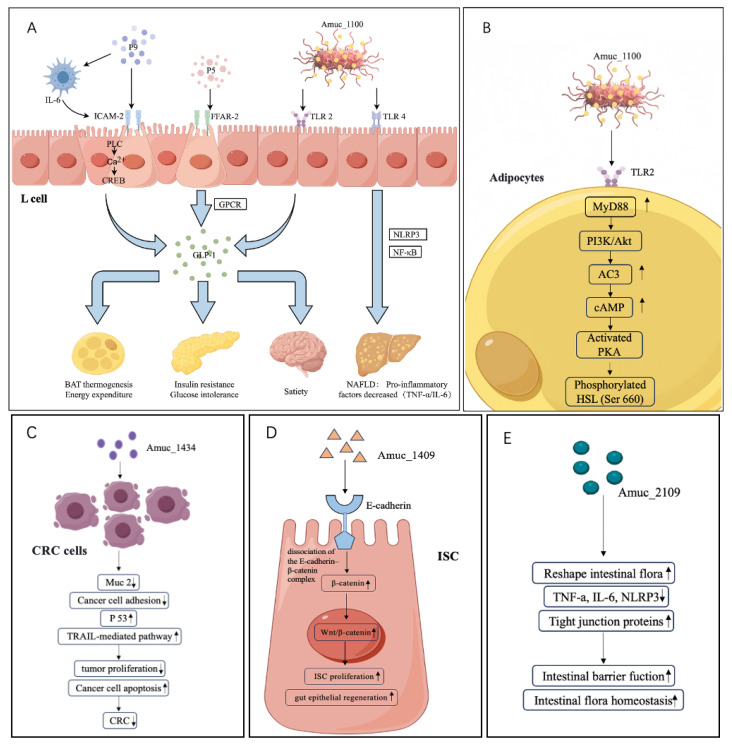
Schematic diagram of Molecular Mechanisms of Disease-Associated Proteins from *A. muciniphila* (**A**) Signaling cascades in intestinal L cells and downstream systemic metabolic effects. GLP-1 secretory pathways: (1) P9 binds to ICAM-2 and synergizes with IL-6 from immune cells to activate the PLC-Ca^2+^-CREM axis, promoting GLP-1 release; (2) P5 activates FFAR-2 (a G protein-coupled receptor, GPCR) to induce GLP-1 synthesis; (3) Amuc_1100 engages TLR2 to augment GLP-1 secretion. Systemic physiological effects of GLP-1: Circulating GLP-1 exerts pleiotropic metabolic effects, including inducing satiety in the brain, improving insulin resistance and glucose intolerance in the pancreas, and promoting thermogenesis and energy expenditure in brown adipose tissue (BAT). Inflammatory suppression pathway: Amuc_1100 alleviates HFD-induced NAFLD by downregulating nucleotide-binding oligomerization NLRP3, TLR4, NF-κB, and pro-inflammatory cytokines (TNF-α, IL-6). (**B**) In adipocytes, Amuc_1100 activates the TLR2–MyD88–PI3K/Akt–AC3 cascade, elevating cAMP to activate PKA, which phosphorylates HSL (Ser 660) to drive lipolysis. (**C**) Amuc_1434 downregulates Muc2 expression in CRC cells to inhibit cancer cell adhesion, upregulates p53 and activates the TRAIL-mediated apoptotic pathway, thereby suppressing tumor proliferation, promoting cancer cell apoptosis, and ultimately blocking CRC progression. (**D**) Amuc_1409 binds to E-cadherin on intestinal epithelial cells, induces dissociation of the E-cadherin/β-catenin complex, activates the Wnt/β-catenin signaling pathway, and promotes intestinal stem cell (ISC) proliferation and gut epithelial regeneration. (**E**) Amuc_2109 reshapes the intestinal flora, downregulates pro-inflammatory factors (TNF-α, IL-6) and NLRP3 inflammasome expression, upregulates tight junction proteins, enhances intestinal barrier function, and maintains intestinal flora homeostasis. Arrows indicate upregulation (↑) or downregulation (↓) of gene/protein expression or biological activity.

Amuc_1100 effectively ameliorates metabolic disturbances induced by a high-fat diet (HFD), including excessive weight gain and systemic inflammation [19]. It promotes lipolysis, adipose tissue browning, and energy expenditure by activating the AC3/PKA/HSL signaling pathway (Figure 2) and upregulates related functional genes. During the differentiation of 3T3-L1 preadipocytes, Amuc_1100 treatment significantly reduces lipid droplet formation [16]. and fat accumulation while stimulating the expression of browning markers.

In addition, Amuc_1100 activates Toll-like receptor 2 (TLR2) to enhance intestinal barrier function [20,21]. and interacts with bile acid signaling [22,23] to reduce body weight and lipid accumulation in mice [24,25]. Amuc_1100 also improves HFD-induced non-alcoholic fatty liver disease by downregulating pro-inflammatory factors and modulating gut microbiota homeostasis [26] (Figure 1).

#### 2.1.2. Amuc_1100 and Immune Regulation

Amuc_1100 primarily exerts its immunomodulatory effects through activation of TLR2. It has been shown to directly bind human TLR2 (hTLR2), leading to activation of downstream NF-κB signaling (Figure 1). This activation promotes the production of the anti-inflammatory cytokine IL-10 while suppressing the expression of pro-inflammatory cytokines, including TNF-α, IL-1β, IFN-γ, and IL-6 [27]. In murine models of acute pancreatitis, pretreatment with Amuc_1100 significantly alleviates pancreatic injury and reduces serum amylase and lipase levels, effects that are associated with inhibition of NF-κB signaling and reduced inflammatory cell infiltration.

#### 2.1.3. Amuc_1100 and Intestinal Protection

Amuc_1100 improves intestinal barrier function through multiple mechanisms [28]: activating CREBH to regulate tight junction protein expression, promoting goblet cell proliferation and mucin secretion, and modulating intestinal stem cell (ISC) activity via the Wnt/β-catenin signaling pathway [7,29] (Figure 1).

#### 2.1.4. Additional Regulatory Functions of Amuc_1100

Amuc_1100 also regulates neurotransmitter metabolism. It has been shown to enhance the expression of tryptophan hydroxylase 1 (Tph1), the rate-limiting enzyme for serotonin (5-hydroxytryptamine, 5-HT) synthesis in enterochromaffin cells, while reducing the expression of the serotonin reuptake transporter (SERT) in intestinal epithelial cells, resulting in increased levels of 5-HT in both the gut and circulation [30] (Figure 1). This finding reveals a novel mechanism by which *A. muciniphila* influences nervous system function via modulation of the gut–brain axis [27].

### 2.2. P9: A Key Factor Regulating GLP-1 Secretion

The discovery of secreted proteins from *A. muciniphila* has largely benefited from advances in proteomic technologies. In 2021, Yoon et al. [8] identified an 84 kDa secreted protein from *A. muciniphila* culture supernatants using fast protein liquid chromatography combined with liquid chromatography-mass spectrometry, and named it P9, encoded by the gene *Amuc_1631*. The identification of P9 filled a major gap in the study of *A. muciniphila* secreted proteins and demonstrated that, in addition to outer membrane proteins, *A. muciniphila* can exert health-promoting effects through secreted factors (Table 1).

P9 belongs to the peptidase S41A family and represents the second *A. muciniphila*-derived functional protein to be extensively characterized after Amuc_1100 [8]. Its most prominent biological activity is the robust induction of glucagon-like peptide-1 (GLP-1) secretion, highlighting its considerable therapeutic potential for the treatment of diabetes and obesity (Figure 1).

P9 induces GLP-1 secretion by binding to its specific receptor intercellular adhesion molecule-2 (ICAM-2) and activating downstream signaling pathways [8]. Notably, the GLP-1–inducing effect of P9 is stronger than that of short-chain fatty acids (SCFAs) and is mediated through a distinct mechanism, indicating unique biological properties.

Beyond GLP-1 induction, P9 also enhances thermogenesis in brown adipose tissue. In HFD-induced obese mice, oral administration of P9 for eight weeks significantly reduces body weight gain, fat mass, and food intake, while increasing brown adipose tissue mass and the expression of thermogenesis-related genes [8]. Indirect calorimetry analyses reveal a reduced respiratory quotient and increased fatty acid oxidation in P9-treated mice, indicating a metabolic shift toward lipid utilization.

The metabolic effects of P9 are dependent on interleukin-6 (IL-6) [8]. In IL-6 knockout mice, the beneficial effects of P9 on glucose homeostasis are completely abolished, accompanied by downregulation of ICAM-2 expression. These findings suggest that IL-6 is a critical mediator of P9 activity and may participate in regulating ICAM-2 expression, forming a positive IL-6–ICAM-2–P9 feedback loop [8].

To facilitate industrial application, P9 has been successfully heterologously expressed in *Lactococcus lactis*. Recombinant *Lactococcus lactis* NZP9 secretes P9 that stimulates GLP-1 secretion in NCI-H716 cells more effectively than the native *A. muciniphila* BAA-835 strain [31], laying the foundation for the development of P9-based probiotic formulations.

### 2.3. Amuc_1409: A Novel Regulator of Intestinal Stem Cells

In 2024, Kang et al. [11] identified another important secreted protein, Amuc_1409, in the cell-free supernatant of *A. muciniphila* cultures (Table 1). Amuc_1409 is the most abundant protein in the *A. muciniphila* secretome and is stably expressed under various culture conditions. Western blotting and nLC-MS/MS analyses revealed that Amuc_1409 is predominantly present in the supernatant, with minor localization in the outer membrane (OM) and periplasmic space (PP). This distribution suggests that Amuc_1409 may be transiently retained during transport from the periplasm to the outer membrane via a non-classical secretion pathway (Figure 1).

Amuc_1409 is a ~40 kDa protein with distinct biological functions, primarily promoting intestinal health through regulation of intestinal stem cell (ISC) activity [11]. It exerts this effect by binding directly to the extracellular domain of E-cadherin and activating the Wnt/β-catenin signaling pathway. Co-immunoprecipitation experiments confirmed that Amuc_1409 disrupts the interaction between E-cadherin and β-catenin in HT-29 cells.

Functionally, Amuc_1409 significantly enhances ISC proliferation and intestinal regeneration [11]. In intestinal organoid cultures, Amuc_1409 treatment markedly increases organoid size and branching in both mouse and human models and upregulates proliferation markers such as Ki67, ASCL2, and OLFM4. In vivo, Amuc_1409 protects mice from radiation- or 5-fluorouracil (5-FU)–induced intestinal injury, extending survival after radiation exposure from 14 to 18 days and alleviating 5-FU–induced weight loss, diarrhea, and histopathological damage.

Notably, Amuc_1409 also exhibits anti-aging effects. In aged mouse models, oral administration of Amuc_1409 for 15 weeks restores age-related declines in ISC number and function, increases ISC and proliferation marker expression in the small intestine, and enhances budding efficiency and branching complexity in aged intestinal organoids [11]. These findings suggest that Amuc_1409 may serve as a novel therapeutic candidate for preventing and treating age-associated intestinal dysfunction [32].

### 2.4. Other Bioactive Proteins with Disease-Modulating Functions

In addition to the major proteins described above, *A. muciniphila* produces several other bioactive proteins with disease-modulating potential. Proteomic analyses have identified additional proteins with putative functions. For instance, P5 has been shown to induce GLP-1 secretion, although with lower potency than P9 [12] (Table 1).

Amuc_1434 is an aspartic protease [33] that suppresses the viability of human colorectal cancer LS174T cells through tumor necrosis factor–related apoptosis-inducing ligand (TRAIL)-mediated apoptotic pathways [9], suggesting a potential role in colorectal cancer prevention and therapy (Table 1).

Amuc_2109, a β-N-acetylhexosaminidase, protects mice from dextran sulfate sodium (DSS)-induced colitis by enhancing intestinal barrier integrity and modulating gut microbiota composition (Table 1). Qian et al. [10] demonstrated that Amuc_2109 ameliorates DSS-induced colitis through four mechanisms: repairing the intestinal barrier, inhibiting oxidative stress, blocking inflammatory signaling pathways, and regulating the gut microbiota.

In 2024, another outer membrane protein, Amuc_1098, was identified. According to patent information, Amuc_1098 is annotated as a PilQ protein derived from type IV pili and localized to the outer membrane of *A. muciniphila*. Its gene cluster enriched in membrane and cell envelope proteins [14]. Similarly to Amuc_1100, Amuc_1098 exhibits anti-inflammatory properties and alleviates clinical symptoms in murine models of acute pancreatitis [32], reducing systemic inflammation and inflammatory cytokine secretion from pancreatic acinar cells (Table 1).

In addition, the *A. muciniphila* genome contains more than 300 putative protein-coding genes (approximately 11%) involved in mucin degradation, although the functions of most of these proteins remain poorly characterized [34]. These proteins may play important roles in host-microbe interactions and warrant further investigation (Figure 1).

Interestingly, components of the *A. muciniphila* cell envelope also exhibit immunomodulatory activity. In 2022, Bae et al. [35] reported that a15:0-i15:0 phosphatidylethanolamine activates immune signaling via the TLR2-TLR1 heterodimer, preferentially inducing a specific subset of inflammatory cytokines. Although its activity is lower than that of known natural or synthetic TLR2 agonists, low-level stimulation of the TLR2-TLR1 pathway may help reset activation thresholds and maintain immune signaling homeostasis.

## 3. Molecular Mechanisms of Disease-Associated Proteins from *A. muciniphila*

### 3.1. Interactions with Host Receptors

Specific binding of *A. muciniphila* proteins to host receptors represents the initial step in the execution of their biological functions. To date, several key receptors have been identified, including TLR2, intercellular adhesion molecule-2 (ICAM-2), and E-cadherin. These receptors not only mediate the direct effects of *A. muciniphila* proteins but also participate in the regulation of downstream signaling cascades.

TLR2 is the primary receptor for Amuc_1100, and their interaction exhibits a high degree of specificity. Structural studies by Wang et al. [13] demonstrated that the extracellular domain of Amuc_1100 forms a stable complex with the ligand-binding domain of TLR2. Notably, the oligomeric state of Amuc_1100 influences its binding affinity for TLR2, with dimeric Amuc_1100 exhibiting stronger TLR2 binding than the monomeric form. This finding suggests that modulation of Amuc_1100 oligomerization may represent an effective strategy for enhancing its biological activity (Figure 2).

ICAM-2 has been identified as the specific receptor for P9, a discovery with important biological implications. ICAM-2 belongs to the immunoglobulin superfamily [36] and is traditionally regarded as a cell adhesion molecule involved in leukocyte trafficking and T cell activation. The identification of ICAM-2 as a P9 receptor revealed a previously unrecognized signaling function. Yoon et al. [8] demonstrated that P9 binds ICAM-2 in a dose-dependent manner, and that ICAM-2–derived peptides or neutralizing antibodies significantly inhibit P9-induced GLP-1 secretion (Figure 2).

E-cadherin serves as the receptor for Amuc_1409, and this interaction displays unique molecular characteristics. Amuc_1409 selectively binds to the extracellular domain of E-cadherin, but not its intracellular domain. This selective interaction induces E-cadherin internalization and β-catenin release, thereby activating the Wnt signaling pathway [11]. Importantly, other *A. muciniphila* proteins, such as P9 and Amuc_1100, do not bind E-cadherin, underscoring the high specificity of this interaction.

Beyond these identified receptors, *A. muciniphila* proteins may interact with additional, as-yet-uncharacterized host receptors. For example, during receptor screening for P9, kinectin 1 (KTN1) was identified as a potential interacting protein [8]. These findings suggest that *A. muciniphila* proteins may exert pleiotropic effects through interactions with multiple host receptors.

### 3.2. Activation and Regulation of Signaling Pathways

The activation and modulation of key intracellular signaling pathways constitute the central mechanisms by which *A. muciniphila* proteins exert their biological effects. Major pathways identified to date include the TLR2 signaling pathway [37], ICAM-2–associated signaling [36], the Wnt/β-catenin pathway [38], and the AC3/PKA/HSL pathway [39]. These pathways intersect to form a complex regulatory network that collectively mediates the health-promoting effects of *A. muciniphila* proteins.

#### 3.2.1. TLR2 Signaling Pathway

The TLR2 signaling pathway is the principal route through which Amuc_1100 exerts its biological effects. Amuc_1100 directly binds TLR2, leading to activation of downstream NF-κB and MAPK signaling cascades. This activation upregulates protective tight junction proteins, including occludin, ZO-1, and claudins-1/3/5/8, while suppressing the expression of the leaky tight junction protein claudin-2 [40]. In addition, Amuc_1100 activates the CREBH–miR-143/145 axis to enhance insulin-like growth factor-1 (IGF-1) signaling and promote intestinal epithelial repair [28]. Concurrently, downregulation of cannabinoid receptor 1 (CB1) reduces intestinal permeability, thereby improving systemic metabolic and inflammatory states [7].

In enterochromaffin cells, TLR2 activation by Amuc_1100 leads to upregulation of tryptophan hydroxylase 1 (Tph1), the rate-limiting enzyme for serotonin synthesis [40]. In intestinal epithelial cells, the same signaling pathway drives alterations in tight junction protein expression and enhances barrier integrity. Notably, the TLR2-binding activity of Amuc_1100 is concentration-dependent, suggesting that dosage may critically influence its biological outcomes.

#### 3.2.2. ICAM-2 Signaling Pathway

The ICAM-2 signaling pathway is essential for the biological activity of P9 (Figure 2). The binding of P9 to ICAM-2 triggers a cascade of intracellular signaling events, including activation of phospholipase C (PLC), intracellular Ca^2+^ release, and phosphorylation of cAMP response element-binding protein (CREB) [8]. These signaling events ultimately lead to robust GLP-1 secretion and activation of brown adipose tissue thermogenesis. Interestingly, ICAM-2 has traditionally been regarded as a cell adhesion molecule; studies on P9 represent the first evidence that ICAM-2 can function as a GPCR-like signaling molecule.

#### 3.2.3. Wnt/β-Catenin Signaling Pathway

The Wnt/β-catenin signaling pathway constitutes the core mechanism by which Amuc_1409 regulates intestinal stem cell (ISC) activity. By binding to E-cadherin and promoting dissociation of the E-cadherin/β-catenin complex, Amuc_1409 induces β-catenin nuclear translocation and activation of Wnt target gene transcription [11]. This mechanism not only promotes ISC proliferation and intestinal regeneration but also possibly influences ISC fate determination (Figure 2).

#### 3.2.4. AC3/PKA/HSL Pathway

The AC3/PKA/HSL pathway is a critical mechanism underlying Amuc_1100-mediated lipolysis (Figure 2). Zheng et al. [16] demonstrated that Amuc_1100 upregulates adenylate cyclase 3 (AC3) expression, increases intracellular cAMP levels, activates protein kinase A (PKA), and ultimately induces phosphorylation and activation of hormone-sensitive lipase (HSL) at Ser660. This signaling cascade accelerates triglyceride hydrolysis and promotes lipid catabolism.

### 3.3. Cell-Type-Specific Effects

Proteins derived from *A. muciniphila* exert distinct effects on different host cell types, and this cell-type specificity constitutes a fundamental basis for their multifunctional biological activities. In intestinal enteroendocrine L cells [41], both P9 and Amuc_1100 are capable of inducing glucagon-like peptide-1 (GLP-1) secretion, albeit through different mechanisms. P9 primarily acts via the ICAM-2–Ca^2+^–CREB signaling axis, whereas Amuc_1100 appears to regulate GLP-1 synthesis mainly through activation of the TLR2–NF-κB pathway [8] (Figure 2).

In intestinal epithelial cells, *A. muciniphila* proteins predominantly influence barrier integrity and cellular proliferation. Amuc_1100 upregulates the expression of tight junction proteins such as occludin, claudin-5, and zonula occludens-1 (ZO-1) [7], while downregulating the permeability-associated protein claudin-2. These changes strengthen intercellular junctions and reduce the translocation of harmful luminal substances. In addition, Amuc_1409 promotes epithelial cell proliferation and migration, thereby accelerating intestinal repair following injury [11].

In immune cells, *A. muciniphila* proteins exhibit pronounced anti-inflammatory activities. Amuc_1100 has been shown to modulate macrophage polarization by promoting the differentiation of anti-inflammatory M2 macrophages while suppressing the activation of pro-inflammatory M1 macrophages [42]. In T cells, Amuc_1100 facilitates differentiation toward Th2 and regulatory T (Treg) cells, while inhibiting Th1 and Th17 cell differentiation, collectively contributing to an overall anti-inflammatory immune profile [43].

In adipocytes, Amuc_1100 displays unique metabolic regulatory properties. In 3T3-L1 preadipocytes, Amuc_1100 suppresses the expression of adipogenesis-related genes while enhancing the expression of genes associated with lipolysis and adipose tissue browning [16]. This dual action results in reduced lipid accumulation and increased energy expenditure, providing a mechanistic rationale for its potential application in obesity treatment.

### 3.4. Protein Interaction Networks

During the execution of their biological functions, *A. muciniphila* proteins not only interact with host receptors but may also form complex interaction networks with other *A. muciniphila* proteins or host proteins. Such network effects may amplify or fine-tune the biological activity of individual proteins, resulting in synergistic or antagonistic outcomes.

Among *A. muciniphila* proteins, Amuc_1100 and P9 appear to exhibit synergistic effects. Under certain experimental conditions, combined administration of Amuc_1100 and P9 induces stronger GLP-1 secretion than either protein alone. This synergy may be attributed to activation of distinct signaling pathways: Amuc_1100 primarily signals through TLR2, whereas P9 acts via ICAM-2, with convergence of these pathways leading to signal integration and amplification. In addition, P5 has also been reported to synergize with these proteins; Niu et al. [12] demonstrated that P5 enhances GLP-1 synthesis and secretion by activating G protein–coupled receptor (GPCR) signaling.

At the level of host protein networks, the effects of *A. muciniphila* proteins involve multiple interconnected signaling pathways. For example, P9-induced GLP-1 secretion not only depends on ICAM-2 but also requires the involvement of IL-6 [8]. IL-6 functions both as a downstream mediator of P9 activity and as a potential regulator of ICAM-2 expression, thereby influencing cellular sensitivity to P9 and forming a complex regulatory feedback loop.

## 4. Applications of *A. muciniphila* Bioactive Proteins in Diseases

### 4.1. Metabolic Diseases

Proteins derived from *A. muciniphila* have demonstrated substantial potential in the prevention and treatment of metabolic diseases, particularly type 2 diabetes mellitus (T2DM) and obesity (Table 2). Description of the study by Depommier et al. [44] has shown that pasteurized *A. muciniphila* can significantly improve insulin resistance and reduce fasting blood glucose and glycated hemoglobin levels. In a randomized, double-blind, placebo-controlled trial, 40 overweight or obese insulin-resistant individuals received a daily oral dose of 1 × 10^10^ CFU of viable or pasteurized *A. muciniphila* for three months. The results indicated that the pasteurized *A. muciniphila* group exhibited significant reductions in plasma insulin levels, insulin resistance, and total cholesterol, accompanied by decreases in body weight, fat mass, and hip circumference. In addition, Wang et al. [45] confirmed that pasteurized *A. muciniphila* and its heat-stable outer membrane protein Amuc_1100 attenuate placental inflammation by regulating placental macrophage polarization, thereby improving glucose metabolic disorders and insulin resistance in mice with gestational diabetes mellitus.

#### 4.1.1. Obesity and Insulin Resistance

In the context of obesity, Amuc_1100 exerts its effects through multiple mechanisms. First, it activates the AC3/PKA/HSL signaling pathway (Figure 2), thereby promoting lipolysis. Second, it induces the browning of white adipose tissue, leading to increased energy expenditure. Third, it regulates the secretion of appetite-related hormones, such as glucagon-like peptide-1 (GLP-1), resulting in reduced food intake [16]. In high-fat diet (HFD)-induced obese mouse models, oral administration of Amuc_1100 significantly attenuated body weight gain, reduced fat mass and hepatic lipid accumulation, and improved glucose tolerance and insulin sensitivity. In addition, *A. muciniphila* treatment was shown to decrease plasma levels of inflammatory cytokines, including TNF-α and IL-6, improve endothelial function, and lower blood pressure. It also modulated lipid metabolism by reducing total cholesterol, triglycerides, and low-density lipoprotein cholesterol levels, while increasing high-density lipoprotein cholesterol [46].

Beyond direct administration, Zhang et al. [47] anchored Amuc_1100 on the surface of *Lactococcus lactis* ZHY1 and demonstrated that the recombinant strain AM-ZHY1 reduced hepatic lipid accumulation in zebrafish by inhibiting lipogenesis and significantly decreasing serum levels of aspartate aminotransferase (AST) and alanine aminotransferase (ALT). Notably, the expression of lipolysis-related genes such as ATGL and UCP2 was not significantly altered, suggesting that AM-ZHY1 primarily regulates hepatic lipid accumulation by suppressing lipid synthesis and absorption rather than enhancing lipid breakdown.

The mechanism of the P9 protein in diabetes treatment is particularly well defined. Studies have demonstrated that P9 strongly induces GLP-1 secretion, a key therapeutic target in current diabetes management [8]. Unlike conventional GLP-1 receptor agonists, P9 stimulates GLP-1 secretion by activating intercellular adhesion molecule-2 (ICAM-2) rather than directly binding to the GLP-1 receptor, which may help avoid receptor desensitization. In addition, P9 promotes thermogenesis in brown adipose tissue, thereby increasing energy expenditure and providing added therapeutic value for obesity-associated diabetes (Figure 2).

In addition to P9, P5 enhances GLP-1 synthesis and secretion by activating G protein–coupled receptor (GPCR) signaling pathways, with free fatty acid receptor 2 (FFAR2) identified as a key target mediating its physiological activity [12] (Figure 2). These findings further support the broad application of protein-enriched *A. muciniphila* (P-AKK) in the food industry and provide a solid theoretical basis for its development as a functional or bioactive food ingredient.

Given that pasteurized *A. muciniphila* [48] and Amuc_1100 [7] also exert beneficial effects on glycemic regulation, P9 is not the sole mediator through which *A. muciniphila* modulates glucose homeostasis. However, the precise mechanisms by which P9 stimulates GLP-1 secretion in L cells, as well as whether P9 is specific to *A. muciniphila*, remain unclear. Moreover, it is still unknown whether P9-induced IL-6 production during GLP-1 stimulation promotes intestinal inflammation or how P9 influences intestinal barrier function (Figure 2).

#### 4.1.2. Non-Alcoholic Fatty Liver Disease

Non-alcoholic fatty liver disease (NAFLD) [49] is a chronic liver disease and a hepatic manifestation of metabolic syndrome. Reduced abundance of *A. muciniphila* has been observed in obese mouse models with NAFLD [50]. The development of NAFLD is closely associated with intestinal barrier dysfunction and dysregulation of the gut-liver axis [51]. Qu et al. [26] demonstrated that Amuc_1100 alleviates HFD-induced NAFLD by downregulating nucleotide-binding oligomerization domain-like receptor protein 3 (NLRP3), TLR4 (Figure 2), NF-κB, and pro-inflammatory cytokines, while also restoring gut microbiota homeostasis.

### 4.2. Inflammatory Diseases

#### 4.2.1. Inflammatory Bowel Disease

Inflammatory bowel disease (IBD) [52], including ulcerative colitis and Crohn’s disease, is characterized by chronic and relapsing intestinal inflammation. *A. muciniphila* proteins have shown significant therapeutic potential in IBD, primarily through anti-inflammatory effects, immune regulation, and protection of the intestinal barrier. Wang et al. [53] reported that *A. muciniphila* and its protein components markedly ameliorated dextran sulfate sodium (DSS)-induced colitis in mice, as evidenced by reduced colon shortening, improved histopathological damage, and decreased inflammatory cell infiltration (Table 3).

Mechanistically, Amuc_1100 exerts anti-inflammatory effects via activation of TLR2. It promotes the production of the anti-inflammatory cytokine IL-10 while suppressing the expression of pro-inflammatory cytokines, including TNF-α, IFN-γ, IL-1β, IL-6, IL-18, and IL-33 [53]. Flow cytometric analysis further revealed that Amuc_1100 treatment significantly reduced the number of CD16/32^+^ macrophages [55] and CD8^+^ cytotoxic T lymphocytes in the colon [56], as well as CD16/32^+^ macrophages in the spleen and mesenteric lymph nodes [53].

Regarding intestinal barrier repair, Kang et al. identified the active protein Amuc_1409 using nLC-MS/MS analysis and demonstrated that it promotes intestinal stem cell proliferation by activating the E-cadherin/β-catenin pathway [11], thereby repairing intestinal barrier damage caused by radiotherapy, chemotherapy, or aging. Additionally, the β-galactosidase Amuc_1686 functions as an altruistic enzyme that is regulated by phospholipids and avoids degradation of the mucus layer covering apoptotic epithelial cells, facilitating epithelial regeneration and barrier repair [57]. In inflamed regions, the β-N-acetylhexosaminidase Amuc_2109 improves intestinal barrier integrity and alleviates DSS-induced colitis by downregulating NLRP3 and pro-inflammatory cytokines [10]. Furthermore, the MbpA-like adhesin Amuc_1620 promotes *A. muciniphila* adhesion to N-acetylglucosamine residues in mucin, thereby facilitating intestinal barrier repair and improving colitis [58].

#### 4.2.2. Pancreatitis

Beyond IBD, *A. muciniphila* proteins have shown therapeutic potential in other inflammatory diseases. In acute pancreatitis models, Amuc_1100 pretreatment significantly attenuated pancreatic injury and reduced serum amylase [59] and lipase [27] levels (Table 3). These protective effects are mediated by inhibition of the NF-κB signaling pathway, reduced inflammatory cytokine expression, decreased infiltration of Ly6C^+^ macrophages and neutrophils, and modulation of gut microbiota composition and tryptophan metabolism. Microbiota analysis revealed that Amuc_1100 pretreatment reduced the abundance of *Bacteroidetes* [60], *Proteobacteria* [61], *Desulfobacterota* [62], and *Campylobacterota* [63], while increasing the proportions of *Firmicutes* [64] and *Actinobacteria* [65].

#### 4.2.3. Hepatic Inflammation

In studies of alcoholic liver disease [66], pasteurized *A. muciniphila* and Amuc_1100 were shown to ameliorate hepatic inflammation and steatosis by modulating gut microbiota composition and host metabolism (Table 3). In 2024, Cheng et al. [54] demonstrated that these interventions reduce intestinal permeability, alleviate endotoxemia [67], suppress hepatic inflammatory responses, and improve lipid metabolism, providing new therapeutic insights for alcoholic liver disease.

#### 4.2.4. Other Inflammatory Conditions

The relative abundance of *A. muciniphila* is significantly reduced in patients with sepsis [68]. Supplementation with live *A. muciniphila* or its fermentation products markedly decreased sepsis-induced mortality. Xie et al. [69] identified a novel tripeptide, Arg-Lys-His (RKH), produced by live *A. muciniphila* using metabolomic analysis. In mouse models, RKH significantly reduced sepsis-induced inflammatory cell activation and excessive production of pro-inflammatory cytokines, thereby protecting against sepsis-related mortality and organ damage. Mechanistic studies revealed that RKH directly binds to TLR4 and blocks TLR4 signaling in immune cells, suggesting that it may function as a novel endogenous TLR4 antagonist. Upon successful clinical translation, RKH could represent a promising therapeutic candidate for the treatment of lethal sepsis (Table 3).

In addition, Mulhall et al. [70] reported that *A. muciniphila* is deficient in patients with periodontitis, and supplementation with *A. muciniphila* or Amuc_1100 alleviated Porphyromonas gingivalis–induced experimental periodontitis. Amuc_1100 was further shown to improve experimental periodontitis by promoting macrophage M2 polarization [42] and upregulating IL-10 expression [71].

### 4.3. Neurological Diseases: Alzheimer’s Disease, Parkinson’s Disease, and Depression

The concept of the “microbiota–gut–brain axis” was proposed [72] more than a decade ago, and accumulating evidence [73] has linked gut microbiota dysbiosis to the pathogenesis of central nervous system (CNS) disorders. This paradigm has provided novel insights into disease mechanisms, biomarker discovery, and potential therapeutic strategies for debilitating neurological diseases (Table 4).

*A. muciniphila* proteins influence neurological disorders through multiple mechanisms. For instance, Amuc_1100 modulates the serotonin (5-hydroxytryptamine, 5-HT) pathway, which is of particular relevance to depression [74]. Studies have shown that Amuc_1100 promotes 5-HT synthesis in enterochromaffin cells while reducing its reuptake, thereby increasing 5-HT levels in the intestine and circulation. Given that approximately 90% of 5-HT is produced in the gut, this regulation may affect CNS function via the gut–brain axis.

In Alzheimer’s disease (AD) models [75,76], supplementation with *A. muciniphila* improved spatial learning and memory deficits in APP/PS1 mice and delayed neuropathological progression [77]. Mechanistically, *A. muciniphila* reduced cerebral β-amyloid (Aβ) deposition, improved glucose metabolism, and protected blood–brain barrier integrity, although the specific active proteins involved remain to be identified.

In models of depression and anxiety, *A. muciniphila* and its protein components exhibit notable antidepressant and anxiolytic activities [74]. In chronic unpredictable mild stress (CUMS)-induced depressive mouse models, oral administration of Amuc_1100 significantly alleviated depression-like behaviors. These effects were associated with increased expression of 5-HT and brain-derived neurotrophic factor (BDNF) [75,78] in the hippocampus and reduced hippocampal inflammatory cytokine levels. Beyond the wild-type protein, structurally modified variants of Amuc_1100 also influence host physiology. In 2022, Cheng et al. [79] reported that deletion of the N-terminal 80 amino acids of Amuc_1100 generated Amuc_1100Δ^80^, which exhibited stronger binding affinity to TLR2 than the wild-type protein. Amuc_1100Δ^80^ activated tryptophan hydroxylase 1 (Tph1) to promote 5-HT synthesis by interacting with TLR2 and reshaping gut microbiota composition, thereby activating the downstream 5-HTR1A–CREB–BDNF signaling pathway. This process suppressed inflammation and hyperactivation of the hypothalamic–pituitary–adrenal axis and alleviated depressive symptoms in mice. In antibiotic-induced anxiety and depression-like behaviors, Amuc_1100 mitigated these effects by modulating the BDNF/TrkB signaling pathway, increasing glial fibrillary acidic protein (GFAP) [80] expression, and reducing c-Fos [81] expression in the hippocampus [82].

Furthermore, *A. muciniphila* has been shown to improve cognitive function, potentially through its effects on neural plasticity. The abundance of *A. muciniphila* declines in aging humans and mice, whereas supplementation alleviates aging-associated cognitive decline via the microbiota–gut–brain axis. He et al. [83] demonstrated that Amuc_1100 improves age-related cognitive impairment by regulating L-arginine metabolism [84], attenuating oxidative stress, and restoring intestinal barrier function.

**Table 4:** The mechanisms and effects of active proteins from *A. muciniphila* in neurological diseases.

Specific Disease	Key Proteins	Experimental Model	Core Mechanisms of Action	Main Effects	Reference
Depression and Anxiety	Amuc_1100	Animal: CUMS-induced depressive mouse models; In vitro: Rat insulinoma cell model (RIN-14B)	Promotes the synthesis and release of 5-HT in enterochromaffin cells; upregulates 5-HT and BDNF expression in the hippocampus	Improves CUMS-induced depressive-like behaviors; relieves antibiotic-induced anxiety and depression; inhibits excessive activation and inflammation of the HPA axis	[74]
	Amuc_1100Δ^80^	Animal: CUMS-induced depressive mouse models; In vitro: Rat insulinoma cell model (RIN-14B)	Enhances TLR2 binding and activates Tph1 to promote 5-HT synthesis; triggers the 5-HTR1A-CREB-BDNF pathway		[79]
Aging-Related Cognitive Impairment	Amuc_1100	Animal: Natural aging mouse models; Senescence-accelerated mouse models; In vitro: Caco-2 cell monolayer model; Primary mouse hippocampal neuron model; Mouse colon organoid model	Regulates L-arginine metabolism; attenuates oxidative stress; restores intestinal barrier function	Improves aging-induced cognitive impairment; delays the aging process via the microbiota-gut–brain axis	[83]

### 4.4. Cancer: Immunotherapy and Tumor Prevention

Cancer immunotherapy represents a major breakthrough in oncology, and *A. muciniphila* proteins have shown remarkable potential in enhancing immunotherapeutic efficacy (Table 5). Notably, the mechanisms underlying their antitumor effects are multifaceted.

In colorectal cancer, previous studies have shown that *A. muciniphila* enhances the activity of CD8^+^ T cells and M1 macrophages, improves the efficacy of PD-1 blockade therapy, and suppresses tumor growth [85]. Meng et al. [86] reported that Amuc_1434, a member of the aspartic protease family, regulates intestinal mucus layer thickness by degrading mucin 2 (Muc2) secreted by LS174T colorectal cancer cells, thereby contributing to restoration of the intestinal barrier. Amuc_1434 arrested the LS174T cell cycle at the G0/G1 phase and upregulated tumor suppressor p53, inhibiting cell proliferation. It also suppressed LS174T cell viability [9] via the TRAIL-mediated apoptotic pathway by increasing mitochondrial reactive oxygen species (ROS) levels and decreasing mitochondrial membrane potential, ultimately promoting apoptosis. These findings suggest a role for Amuc_1434 in controlling colorectal cancer progression. In addition, the acetyltransferase Amuc_2172 contained in extracellular vesicles derived from *A. muciniphila* reprograms the tumor microenvironment by inducing HSP70 secretion and enhancing cytotoxic T lymphocyte–associated immune responses, thereby inhibiting colorectal cancer progression [87].

In lung adenocarcinoma [88], Amuc_1100 enhances antitumor immunity by modulating the immune microenvironment and inhibiting immune evasion. Xu et al. [89] demonstrated that Amuc_1100 significantly accelerated recruitment of CD8^+^ T cells to tumors and increased granzyme B [90] and IFN-γ levels, thereby enhancing CD8^+^ T cell cytotoxicity. Concurrently, Amuc_1100 suppressed tumor immune evasion by reducing PD-L1 expression through inhibition of the JAK–STAT signaling pathway. Moreover, Amuc_1100 epigenetically regulated RNF181 acetylation and ATG7 ubiquitination to activate CD8^+^ T cell–mediated antitumor immunity [91]. Specifically, Amuc_1100 promoted acetylation of the RNF181 promoter, upregulated its expression, mediated ubiquitin-dependent degradation of ATG7, and ultimately downregulated PD-L1 expression, thereby enhancing CD8^+^ T cell antitumor activity.

Beyond direct antitumor effects, *A. muciniphila* proteins also contribute to cancer prevention [92]. By modulating gut microbiota composition, reducing carcinogen production, and strengthening intestinal barrier function [93], Amuc_1100 triggers the activation of TLR2 signaling, which not only upregulates the expression of tight junction components (occludin, claudin-1 and ZO-1) to reduce intestinal mucosal permeability and limit systemic translocation of endotoxins and carcinogens [7], but also augments the antitumor effector function of CD8^+^ T cells [89]. By reinforcing intestinal barrier integrity and boosting immune surveillance, Amuc_1100 thereby impedes colitis-associated colorectal cancer (CAC) tumorigenesis. In addition, the RKH tripeptide interacts directly with TLR4 to inhibit pro-inflammatory signaling, thereby alleviating the risk of chronic inflammation-driven tumor development [69].

**Table 5:** The mechanisms and effects of active proteins from *A. muciniphila* in cancer.

Specific Disease	Key Proteins	Experimental Model	Core Mechanisms of Action	Main Effects	References
Colorectal Cancer	Amuc_1434	Animal: Colorectal cancer (CRC) xenograft mouse model In vitro: Human colon cancer cell model (LS174T); human cervical cancer cell model (HeLa), Human colorectal cancer cell model (SW480, HT-29), human primary CD8^+^ T cells, mouse hippocampal cell model (HT22)	Degrades Muc2 to repair the mucus layer; blocks the G0/G1 phase of the cell cycle; induces TRAIL-mediated apoptosis	Inhibits colorectal cancer cell proliferation and tumor growth; potentiates the efficacy of anti-PD-1 blockade therapy; remodels the tumor microenvironment to promote anti-tumor immunity	[9,86]
Lung Adenocarcinoma	Amuc_1100	Animal: Lewis lung carcinoma subcutaneous allograft mouse model; In vitro: Human lung adenocarcinoma cell model (A549, NCI-H1395); Primary CD8^+^ T cell model	Recruits CD8^+^ T cells and enhances their cytotoxicity (increases granzyme B and IFN-γ levels); inhibits the JAK-STAT pathway to reduce PD-L1 expression; epigenetically regulates RNF181 acetylation and ATG7 ubiquitination	Enhances anti-tumor immune responses; inhibits tumor immune evasion; activates the anti-tumor activity of CD8^+^ T cells	[89]

## 5. Clinical Translational Prospects and Industrial Development

### 5.1. Progress in Clinical Trials and Safety Evaluation

Currently, clinical trials targeting *A. muciniphila*–derived proteins are being actively conducted across multiple countries and regions, with a primary focus on the prevention and treatment of metabolic diseases. It is important to emphasize that the current human clinical evidence regarding *A. muciniphila* is still in the preliminary stage. The most representative human study to date is an exploratory proof-of-concept trial conducted in Belgium, which randomly assigned 40 overweight or obese insulin-resistant subjects to three groups. Two intervention groups received a daily oral dose of 1 × 10^10^ CFU of viable or pasteurized *A. muciniphila* for a 3-month intervention period, with a relatively small number of completers in each arm (approximately 32 subjects actually completed the trial) [94].

The preliminary results of this trial demonstrated that, compared with the placebo group, supplementation with pasteurized *A. muciniphila* was associated with a significant reduction in plasma insulin levels, insulin resistance, and total cholesterol. In addition, it could decrease leukocyte counts and circulating lipopolysaccharide levels, as well as moderately reduce body weight, fat mass, and hip circumference. Importantly, 3-month continuous supplementation with pasteurized *A. muciniphila* significantly reduced the activity of dipeptidyl peptidase-4 (DPP-4), a finding that may be potentially beneficial for diabetes management. Notably, despite the improvements in metabolic indicators in this cohort, no significant changes were observed in plasma GLP-1 levels in the trial.

The aforementioned exploratory trial indicated that both live and pasteurized *A. muciniphila* exhibited preliminary favorable safety and tolerability profiles during the 3-month intervention period, with no serious adverse events reported. Hematological and biochemical analyses showed no adverse effects on liver or kidney function or routine blood parameters. Notably, supplementation with *A. muciniphila* did not significantly alter the overall structure of the gut microbiota, suggesting preliminary good ecological safety. It should be noted that these safety results are based on a small sample size and short intervention duration, and long-term safety data remain to be supplemented.

### 5.2. Product Development and Market Prospects

Translational research on *A. muciniphila* has driven the development of related products, with formulations primarily categorized into live bacterial preparations, pasteurized bacterial formulations, and protein extracts. Pasteurized *A. muciniphila* preparations, in particular, have garnered increasing attention due to their superior stability, safety profile, and consistent biological efficacy—advantages that address key challenges in probiotic delivery and application, laying a foundation for industrial translation.

Against this backdrop, industrial interest in translating *A. muciniphila* research into practical applications has intensified, as evidenced by verifiable industry actions. In June 2025, Danone acquired The Akkermansia Company (TAC), a Belgium-based entity specializing in next-generation probiotics, to strengthen its capabilities in gut health and microbiome research. This acquisition serves as a clear signal of growing investment and resource allocation by major food and beverage corporations in the translational potential of *A. muciniphila*, aligning with the expanding scientific recognition of its role in metabolic and gut health [95].

### 5.3. Regulatory Status (EU/UK/US)

The majority of current studies are based on in vitro experiments and animal models, and their relevance to human physiology remains to be fully validated. Notably, the European Food Safety Authority (EFSA) issued a scientific opinion in 2021 supporting the safety of pasteurized *A. muciniphila* as a novel food under specific conditions, rather than a general approval [96].

In the EU, EFSA’s 2021 scientific opinion defines the initial safe use of pasteurized *A. muciniphila* for adults (excluding pregnant/lactating women) in food supplements and special medical foods, with a confirmed safe daily dose of 3.4 × 10^10^ cells/day (equivalent to 34 billion TFU). Extensions of use approved in 2025–2026 include adolescents aged 12 to <14 years (up to 2.1 × 10^10^ cells/day) and 14 to <18 years (up to 3.0 × 10^10^ cells/day), while safety in pregnant and lactating women and children under 12 years old remains unestablished [97,98]. In the UK, the Food Standards Agency (FSA) and Food Standards Scotland (FSS) approved pasteurized *A. muciniphila* as a novel food in 2023 for use in food supplements and special medical foods for individuals aged 12 years and above, with a maximum assessed dose of 4 × 10^10^ cells/day [99]. In the US, the Food and Drug Administration (FDA) has acknowledged New Dietary Ingredient (NDI) submissions for pasteurized *A. muciniphila*, with approved doses of 1.0 × 10^10^ cells/day (NDI 1363) and 3.0 × 10^10^ cells/day (NDI 1438) for dietary supplement use [100,101].

Moreover, some studies have administered doses of Amuc_1100 as high as 100 μg per day in mice, exceeding physiological concentrations in the normal gut. Future research should investigate potential dose-dependent effects of individual proteins across different diseases and determine appropriate therapeutic dosing regimens aligned with established regulatory dose limits where applicable.

## 6. Controversies

The relationship between *A. muciniphila* and colitis remains one of the most intriguing paradoxes in gut microbiome research, with findings oscillating dramatically between protective and pathogenic roles, contingent upon host context, microbial factors, and environmental conditions. This controversy extends beyond colitis to encompass infection susceptibility, colorectal cancer development, and even metabolic and neurological disorders, highlighting the bacterium’s chameleonic nature in host health and disease.

In conventional DSS-induced colitis models, *A. muciniphila* supplementation consistently demonstrates protective effects, attributed to upregulation of tight junction proteins (ZO-1, Occludin) for gut barrier repair, mitigation of endoplasmic reticulum stress, and promotion of regulatory T cell (Treg) responses. Luo et al.’s comprehensive review [102] further corroborates that live *A. muciniphila* enhances tight junction protein expression, reinforcing intestinal barrier integrity. Similarly, the *A. muciniphila* surface protein Amuc_1100 recapitulates these protective effects, suggesting structural components may mediate therapeutic potential independent of viable bacteria.

However, this beneficial profile undergoes a striking reversal in genetically susceptible hosts. In IL-10 knockout mice, a model of spontaneous colitis, monocolonization with *A. muciniphila* exacerbates inflammation, thinning the mucus layer and elevating pro-inflammatory cytokines. This phenotype highlights how *A. muciniphila*’s defining mucin-degrading activity transforms from a symbiotic adaptation to a pathological liability when host anti-inflammatory capacity is compromised. The host protein Intelectin-1 (ITLN1) introduces another layer of complexity: Grant et al. [6] demonstrated that ITLN1, overexpressed in ulcerative colitis (UC) patients, specifically binds *A. muciniphila* and recruits it to the epithelial surface, intensifying immune activation and tissue damage in inflammatory environments. This host-mediated “hijacking” converts *A. muciniphila* from a potential commensal to a pro-inflammatory accomplice, dependent on disease context.

The controversy extends to infection dynamics, where *A. muciniphila*’s role shifts with dietary fiber availability [103]. In the presence of adequate fiber, *A. muciniphila* produces protective metabolites like the tripeptide RKH [69], which blocks TLR4 signaling to prevent lethal sepsis, and Amuc_1100, which safeguards against Salmonella-induced liver injury. Conversely, fiber deprivation triggers *A. muciniphila*’s “mucus-eating” behavior, compromising the intestinal barrier and facilitating lethal Citrobacter rodentium infection—a phenotype reversed by *A. muciniphila* depletion. Restoring fiber reverses this effect, with *A. muciniphila* then correlating with reduced pathogen burden, underscoring nutrition as a critical determinant of its functional identity.

In CRC, *A. muciniphila* presents another contradictory profile [104]. While some studies report *A. muciniphila* enrichment in CRC tissues and mouse models, implying potential procarcinogenic activity via mucus barrier degradation, others demonstrate tumor-suppressive effects through extracellular vesicles (EVs) and the acetyltransferase Amuc_2172 [87]. The most consistent finding emerges in cancer immunotherapy, where *A. muciniphila* abundance correlates with improved response to PD-1 checkpoint inhibitors across multiple malignancies, with fecal microbiota transplantation studies confirming *A. muciniphila*’s capacity to restore treatment sensitivity by modulating systemic immune responses.

Beyond intestinal inflammation, *A. muciniphila*’s controversial roles persist in metabolic and neurological diseases. In metabolic disorders, evidence consistently supports *A. muciniphila*’s beneficial impact, with both live and pasteurized *A. muciniphila* improving insulin sensitivity, reducing cholesterol, and limiting fat accumulation through GLP-1 secretion and fatty acid metabolism regulation. In contrast, neurological conditions like Parkinson’s disease and multiple sclerosis present conflicting associations: *A. muciniphila* often enriches in patient microbiomes, with in vitro studies suggesting potential to induce α-synuclein aggregation or pro-inflammatory responses, yet some reports link higher *A. muciniphila* levels to reduced disability in multiple sclerosis, proposing a compensatory protective mechanism. The role of *A. muciniphila* in Parkinson’s disease (PD) [76] appears to be complex [105]. While some studies report reduced abundance of *A. muciniphila* in fecal samples from patients with Parkinson’s disease, others have observed significant increases [106], potentially reflecting differences in disease stage, medication use, or individual variability [107]. Nevertheless, given its ability to modulate gut microbiota composition, enhance intestinal barrier function, and reduce inflammation, *A. muciniphila* may exert beneficial effects on PD symptoms.

Resolving these controversies is imperative for translational applications, as indiscriminate *A. muciniphila* supplementation could yield adverse outcomes in susceptible individuals. Future research must clarify context-dependent mechanisms, identify therapeutic strains, and develop precision approaches that account for host-specific factors to harness *A. muciniphila*’s benefits while mitigating risks in colitis and beyond.

## 7. Perspectives and Outlook

Disruption of gut homeostasis has been recognized as a common pathological basis of systemic diseases, while the gut–brain axis serves as a central communication network linking the intestine and the brain and plays a critical role in disease onset and progression. Within the gut microbiota, *A. muciniphila* occupies a pivotal position. Accumulating evidence indicates that this species profoundly influences multiple aspects of host health, including metabolic regulation, inflammatory responses, and the pathogenesis of neuropsychiatric disorders. Through functions such as enhancement of intestinal barrier integrity, efficient mucin degradation, and production of short-chain fatty acids, *A. muciniphila* has established itself as a key determinant of gut health and beyond, making it a representative next-generation probiotic. The diverse functional proteins produced by *A. muciniphila* exhibit remarkable potential for applications in human health. Nevertheless, despite these promising findings, significant challenges remain in translating basic research into practical clinical applications.

The majority of current studies are based on in vitro experiments and animal models, and their relevance to human physiology remains to be fully validated. Although *A. muciniphila*–derived active proteins have demonstrated efficacy across a wide range of diseases, potential mechanistic links among these conditions remain incompletely understood. For example, emerging evidence suggests associations between *A. muciniphila* and neurodegenerative or neuropsychiatric disorders, and Amuc_1100 has shown therapeutic promise in depression and anxiety [108]. However, its role in Parkinson’s disease and the underlying mechanisms require further clarification.

This review summarizes current advances in the study of disease-associated proteins derived from *A. muciniphila* and provides an in-depth analysis of their molecular mechanisms and clinical translational prospects. The main conclusions are as follows:

First, regarding protein discovery and identification, the disease-associated proteins of *A. muciniphila* reported to date include outer membrane proteins Amuc_1100, and Amuc_1098, as well as secreted proteins such as P9 (Amuc_1631), P5, Amuc_1409, Amuc_1434, and Amuc_2109. These proteins have been successfully identified using proteomic approaches, and their biological functions have been validated in multiple in vitro and in vivo models. In particular, in-depth investigations of Amuc_1100, P9, and Amuc_1409 have provided critical insights into the mechanisms underlying *A. muciniphila*–mediated host regulation.

Second, at the molecular level, *A. muciniphila* proteins exert their effects by activating multiple signaling pathways, including TLR2, ICAM-2, Wnt/β-catenin, and AC3/PKA/HSL. These pathways interact to form a complex regulatory network that enables *A. muciniphila* proteins to participate in metabolic regulation, immune modulation, and intestinal protection. Notably, the receptor specificity and mechanisms of action differ among individual proteins, offering opportunities for the development of precision therapeutic strategies.

Third, with respect to disease applications, *A. muciniphila* proteins have demonstrated therapeutic efficacy across a broad spectrum of conditions, including metabolic diseases (obesity, insulin resistance, and NAFLD), inflammatory diseases (IBD, colitis, and pancreatitis), neurological disorders (Alzheimer’s disease and depression), and cancer (immunotherapy enhancement and tumor prevention). This wide disease coverage highlights their value as multifunctional therapeutic molecules. Both live and pasteurized *A. muciniphila* exhibited preliminary good safety and tolerability during the 3-month intervention period, with no serious adverse events reported. It had no adverse effects on liver and kidney function or routine blood parameters, and supplementation with A. muciniphila did not significantly alter the overall structure of the gut microbiota, suggesting preliminary good ecological safety. It should be noted that these safety results are based on a small sample size and short intervention duration, and long-term safety data remain to be supplemented.

Looking ahead, substantial opportunities remain for advancing research and applications of *A. muciniphila* proteins. Future priorities include: (i) identification and characterization of additional functional proteins to expand the *A. muciniphila* proteomic landscape; (ii) elucidation of synergistic interactions among proteins to enable multi-target combination therapies; (iii) strengthening clinical translational research, particularly personalized therapeutic strategies for specific diseases; (iv) advancing industrial technologies, including efficient production, stabilization, and quality control; and (v) enhancing international collaboration to establish globally harmonized standards and accelerate worldwide deployment of *A. muciniphila*–based technologies.

In summary, research on disease-associated proteins derived from *A. muciniphila* opens new avenues for improving human health, with broad prospects in disease prevention and treatment. With continued scientific advances and technological innovation, therapeutic strategies based on *A. muciniphila* proteins are poised to bring transformative benefits to human health.

## Figures and Tables

**Table 1:** Functions of disease-associated proteins from *Akkermansia muciniphila*.

Active Protein Type	Name	Core Biological Functions	Mechanism of Action	Involved Signaling Pathways
Outer membrane protein	Amuc_1100 (PAS)	Intestinal barrier repair, inflammation inhibition, glycolipid metabolism improvement, 5-HT regulation	Binds to TLR2 receptor, activates MAPK and CREBH signaling pathways, inhibits NF-κB	AC3/PKA/HSL, TLR2/NF-κB, CREBH, Wnt/β-catenin
	Amuc_1098	Exerts anti-inflammatory effects and ameliorates symptoms of acute pancreatitis	-	-
Secreted protein	P9 (Amuc_1631)	Potent induction of GLP-1 secretion, anti-obesity, improvement of glucose homeostasis	Binds to ICAM-2 receptor, activates CaMK/CREB signaling pathway, promotes GLP-1 synthesis and secretion	PLC/Ca^2+^/CREB, IL-6-ICAM-2 positive feedback loop
	Amuc_1409	Intestinal stem cell regulation, intestinal regeneration	Binds to the extracellular domain of E-cadherin, promotes dissociation of the E-cadherin/β-catenin complex, and facilitates β-catenin nuclear translocation to activate target genes	Wnt/β-catenin
	P5	Promotes the secretion of GLP-1 from intestinal enteroendocrine L cells in a concentration-dependent manner	Binds to FFAR-2, activates GPCR signaling pathway, promotes the release of GLP-1	-
Other functional proteins	Amuc_1434	Mucin degradation, colorectal cancer inhibition	Muc2 hydrolysis, TRAIL/p53 signaling pathway activation	TRAIL apoptotic pathway
	Amuc_2109	Terminal degradation of mucin O-glycans, intestinal barrier repair, inflammation inhibition	Releases GlcNAc/GalNAc, upregulates tight junction proteins, inhibits NLRP3 inflammasome	-

**Table 2:** The mechanisms and effects of active proteins from *A. muciniphila* in metabolic diseases.

Specific Disease	Key Proteins	Experimental Model	Core Mechanisms of Action	Main Effects	Reference
Obesity and Insulin Resistance	Amuc_1100	Animal: HFD-induced obese mouse model; In vitro: 3T3-L1 preadipocytes; Primary inguinal white adipocytes; BAT primary cells	Activates the AC3/PKA/HSL pathway to promote lipolysis; induces browning of white adipose tissue; regulates GLP-1 secretion	Improves insulin resistance and glucose tolerance; reduces body weight, fat mass and hepatic fat content; regulates lipid metabolism (decreases total cholesterol and triglycerides, increases HDL-C); reduces levels of inflammatory factors (TNF-α, IL-6)	[16,46]
	P9	Animal: HFD-induced C57BL/6J mouse model; IL-6-KO mouse model; In vitro: NCI-H716 cell model; GLUTag cell model; Primary human intestinal epithelial cells; primary brown preadipocytes; HTLA cell model	Activates ICAM-2 to induce GLP-1 secretion; promotes thermogenesis in brown adipose tissue		[8]
	P5	Animal: T2DM mouse model; In vitro: STC-1 cell model	Activates FFAR-2 to enhance GLP-1 synthesis and secretion		[12]
NAFLD	Amuc_1100	Animal: HFD-induced NAFLD mouse model; In vitro: Hepatocyte steatosis model	Downregulates NLRP3, TLR4, NF-κB and pro-inflammatory cytokines; modulates the homeostasis of intestinal flora	Improves HFD-induced hepatic steatosis and inflammation; decreases serum AST and ALT levels; inhibits hepatic lipid synthesis and absorption	[26]

**Table 3:** The mechanisms and effects of active proteins from *A. muciniphila* in Inflammatory diseases.

Specific Disease	Key Proteins	Experimental Model	Core Mechanisms of Action	Main Effects	Reference
IBD	Amuc_1100	Animal: Colitis-associated colorectal cancer (CAC) mouse model; In vitro: CD8^+^ T cell regulation and functional model	Activates TLR2 to promote IL-10 secretion and inhibit pro-inflammatory factors; reduces infiltration of pro-inflammatory immune cells	Ameliorates DSS-induced colitis symptoms (reduces colon shortening and tissue damage); repairs intestinal barrier injury; promotes intestinal epithelial regeneration	[53]
	Amuc_1409	Animal: 5-FU-induced mouse intestinal injury model; In vitro: Mouse small intestinal organoids; Human intestinal organoids; HT-29 cells; HEK293T cells; CT-26 cells	Activates the E-cadherin/β-catenin pathway to promote intestinal stem cell proliferation		[11,32]
	Amuc_2109	Animal: DSS-induced mouse colitis model; In vitro: Caco-2 cell monolayer model; HT-29 colonic epithelial cell model; PMA-differentiated THP-1 human macrophage model	Downregulates NLRP3 and pro-inflammatory factors		[10]
Acute Pancreatitis	Amuc_1100	Animal: Caerulein + LPS-induced acute pancreatitis mouse model; In vitro: AR42J rat pancreatic acinar cell model; RAW264.7 macrophage model	Inhibits the NF-κB pathway to reduce inflammatory factors; modulates intestinal flora and tryptophan metabolism; decreases infiltration of Ly6C^+^ macrophages and neutrophils	Alleviates pancreatic injury; decreases serum amylase and lipase levels; modulates intestinal flora (increases the abundance of Firmicutes/Actinobacteria, decreases Bacteroidetes/Proteobacteria)	[27]
Alcoholic Liver Disease	Amuc_1100	Animal: Chronic-plus-binge ALD model; In vitro: Liver tissue	Reduces intestinal permeability and endotoxemia; inhibits hepatic inflammation; improves lipid metabolism	Attenuates hepatic inflammation and steatosis; modulates intestinal flora and host metabolism	[54]
Periodontitis	Amuc_1100	Animal: Murine experimental periodontitis model; in vitro: Bone marrow–derived macrophage model	Promotes M2 polarization of macrophages; upregulates IL-10 expression	Ameliorates Porphyromonas gingivalis-induced experimental periodontitis; reduces local inflammation	[42]

## Data Availability

No new data were created or analyzed in this study.
